# Topology Resetting During Transcription-Coupled Nucleotide Excision Repair

**DOI:** 10.3390/ijms27167244

**Published:** 2026-08-14

**Authors:** Tae-Hee Lee, Jeseok Jeon, Seo-Gyeong Jo, Tae-Hong Kang

**Affiliations:** 1 Department of Radiation Oncology and Molecular Radiation Sciences, School of Medicine, Johns Hopkins University, Baltimore, MD 21287, USA; tlee164@jhmi.edu; 2 Department of Biomedical Sciences, Dong-A University, Busan 49315, Republic of Korea; 2577615@donga.ac.kr (J.J.); skcho9822@donga.ac.kr (S.-G.J.)

**Keywords:** transcription-coupled nucleotide excision repair, TC-NER, RNA polymerase II, CSB, TOP1, TOP2, DNA supercoiling, chromatin remodeling, R-loops, TFIIH

## Abstract

Transcription-coupled nucleotide excision repair (TC-NER) removes transcription-blocking lesions from active genes, but how lesion-stalled RNA polymerase II (RNAPII) is converted into a repair-accessible substrate remains incompletely understood. Active chromatin is dynamic but not topology-free. RNAPII elongation generates torsional stress that is buffered by nucleosome dynamics, chromatin remodelers, topoisomerases, and gene-body organization. Upon lesion-induced RNAPII arrest, this buffering system may fail locally, creating a topologically and architecturally constrained repair substrate. Here, we integrate established mechanisms of CSB-dependent RNAPII remodeling and recently defined ubiquitin-dependent clearance pathways with a testable model in which local torsional stress and topoisomerase-mediated relaxation influence repair permissiveness. In this framework, CSB remodels and organizes lesion-stalled RNAPII complexes, whereas topoisomerase may contribute to relaxation of transcription-generated supercoiling, thereby promoting a repair-permissive chromatin state. Recent evidence further indicates that CRL4^CSA^-dependent RNAPII ubiquitylation initiates a hierarchical clearance program in which TFIIH/XPD drives rapid RNAPII displacement and VCP/p97 provides a backup extraction pathway. Together, these findings support a model in which TC-NER is possibly licensed not only by repair-factor recruitment but also by coordinated remodeling of RNAPII architecture, chromatin topology, and polymerase fate.

## 1. Introduction

Nucleotide excision repair (NER) removes helix-distorting DNA lesions, including ultraviolet-induced cyclobutane pyrimidine dimers, (6-4) photoproducts, bulky chemical adducts, and certain platinum-induced DNA lesions [1]. In mammalian cells, NER is divided into global genome NER (GG-NER), which surveys the entire genome, and transcription-coupled NER (TC-NER), which preferentially removes transcription-blocking lesions from the transcribed strand of active genes [2]. TC-NER is initiated when elongating RNA polymerase II (RNAPII) encounters a lesion on the template strand and becomes arrested [3]. This stalled RNAPII complex serves as the initial damage sensor and recruits a specialized set of repair-coupling proteins [4]. Upon RNAPII stalling, cockayne syndrome protein B (CSB) binds at the arrested complex, followed by cockayne syndrome protein A (CSA) and UV-stimulated scaffold protein A (UVSSA) assembly, transcription factor IIH (TFIIH) recruitment, DNA opening, lesion verification by xeroderma pigmentosum group A protein (XPA) and replication protein A (RPA), dual incision by xeroderma pigmentosum group G protein (XPG) and xeroderma pigmentosum group F (XPF)-excision repair cross-complementation group 1 (ERCC1), repair synthesis, ligation, and transcription restart [5]. This sequential model has been highly productive, but it does not fully account for the physical state of the DNA and chromatin surrounding stalled RNAPII. Recent time-resolved analyses now add an important fate-control layer to this sequence, showing that RNAPII removal is not a passive consequence of repair assembly but is actively governed by CSA-containing cullin-RING E3 ubiquitin ligase 4 (CRL4^CSA^)-dependent ubiquitylation, xeroderma pigmentosum group D protein (XPD)-driven dissociation, and backup valosin-containing protein (VCP)-dependent extraction [6].

DNA repair occurs in chromatin, where nucleosomes, histone modifications, chromatin remodelers, histone chaperones, DNA-binding proteins, transcription complexes, nascent RNA, DNA supercoiling, and higher-order genome folding all influence accessibility and repair outcome [7]. TC-NER is especially challenging because the lesion is detected by the same RNAPII complex that sterically shields the damaged template strand [8]. Therefore, the early problem in TC-NER is not only molecular recognition but also physical conversion, in which a lesion-stalled transcription complex is transformed into a repair-permissive chromatin intermediate.

Recent studies have emphasized that the functional unit of transcriptional regulation lies between individual nucleosomes and megabase-scale topologically associating domains (TADs) [9]. At this mesoscale level, chromatin is organized as a heterogeneous landscape of local structural units, ranging from nucleosome clutches and nanoscale packing domains to kilobase-scale regulatory microcompartments, enhancer–promoter loops, cohesin-enriched islands, and promoter- or gene-body-associated contact stripes [10]. Many of these structures are dynamic and heterogeneous across single cells, and some persist independently of CCCTC-binding factor (CTCF) or cohesin, whereas others are generated by ongoing transcription itself [11]. Thus, a lesion-stalled RNAPII complex should be viewed not simply as a polymerase arrested on nucleosomal DNA, but as a chromatin-embedded structure positioned within a dynamic local architecture shaped by cohesin-mediated loop extrusion, epigenetically defined interactions, transcriptional condensate-like hubs, nucleosome spacing, and polymerase-driven chromatin motion [12].

This review develops a revised model in which TC-NER is understood as a chromatin-topology resetting pathway. Active promoters and enhancers are frequently characterized by nucleosome-depleted regulatory regions, while transcribed gene bodies are dynamically remodeled as elongating RNAPII traverses, disrupts, and reassembles nucleosomes with the assistance of histone chaperones and chromatin-remodeling factors [13,14]. Under normal conditions, nucleosome breathing, histone chaperones, ATP-dependent chromatin remodelers, and topoisomerases collectively buffer transcription-generated torsional stress, thereby maintaining a chromatin environment permissive for RNAPII elongation [15]. However, active chromatin is not devoid of topological constraints [16,17]. Consistent with the twin-supercoiled-domain model, RNAPII translocation along the DNA helix generates positive supercoiling downstream of the polymerase and negative supercoiling upstream [18,19]. Recent genome-wide measurements have provided direct evidence that transcription-generated DNA supercoiling is broadly present in living human cells. Using azide–trimethylpsoralen sequencing, Yao et al. identified transcription-dependent negative and positive twin-supercoiled domains around active genes and mapped larger supercoiling domains throughout the human genome. These domains were modulated predominantly by TOP1 and TOP2B, demonstrating that transcription-associated topological tension is not restricted to simplified in vitro systems but represents a widespread feature of human chromatin [20]. Upon RNAPII stalling at a bulky lesion, forward translocation is blocked, allowing torsional stress to accumulate beyond the local buffering capacity of chromatin dynamics and topoisomerases [21,22]. The stalled RNAPII complex therefore constitutes both a steric obstacle and a topological barrier, forming a constrained nucleoprotein intermediate that must be remodeled to permit repair-factor access and downstream incision.

In this framework, CSB and DNA topoisomerase I (TOP1) are best viewed as functionally linked regulators of lesion-stalled transcription complexes, with CSB remodeling nucleoprotein architecture and TOP1 modulating the local topological state. CSB is an ATP-dependent SWI2/SNF2-family chromatin-remodeling protein that is recruited to lesion-stalled RNAPII and acts as an early organizer of TC-NER by remodeling the arrested transcription complex and facilitating the recruitment of CSA, UVSSA, TFIIH, and other downstream repair factors [23]. TOP1 relaxes transcription-generated DNA supercoiling through a transient single-strand DNA cleavage–religation cycle that permits controlled rotation of the DNA strand before religation [24,25]. This review addresses how a lesion-stalled RNAPII complex, embedded within active yet topologically constrained chromatin, is physically remodeled into a repair-competent substrate accessible to TFIIH and the canonical NER incision machinery. We propose that CSB and TOP1 may functionally converge on this transition. CSB remodels the nucleoprotein architecture of the arrested complex, whereas TOP1 may tune the local topological state that influences repair permissiveness.

The conceptual novelty of this review lies in treating early TC-NER as a physical substrate-conversion problem rather than solely as a sequential repair-factor recruitment pathway. To our knowledge, this is the first framework to explicitly integrate lesion-stalled RNAPII remodeling, transcription-generated DNA supercoiling, topoisomerase-dependent torsional regulation, mesoscale chromatin organization, and the recently defined hierarchy of RNAPII clearance into a unified model of repair permissiveness. This perspective also introduces the possibility that RNAPII preservation versus clearance may be influenced by the mechanical state of the stalled transcription complex.

## 2. The Unresolved Physical Problem in TC-NER

The initiating event in TC-NER is the stalling of elongating RNAPII at a transcription-blocking DNA lesion on the template strand of an actively transcribed gene. In contrast to GG-NER, where DNA damage is detected primarily by dedicated lesion-recognition factors such as xeroderma pigmentosum group C protein (XPC) [26] and ultraviolet-damaged DNA-binding protein complex (UV-DDB) [27], TC-NER relies on lesion-stalled RNAPII as the initial sensor of transcription-blocking damage. RNAPII stalling therefore serves as a highly selective signal that the transcribed strand harbors a transcription-blocking lesion, thereby linking damage recognition directly to impaired RNA synthesis.

The mammalian TC-NER reaction can be divided into three interconnected phases comprising damage sensing by arrested RNAPII, assembly of a repair-coupling intermediate, and execution of the core NER reaction [28,29]. RNAPII stalls when a bulky lesion interferes with template-strand decoding, nucleotide addition, or forward translocation through the polymerase active site [30]. CSB is subsequently recruited and stabilizes and remodels the arrested elongation complex, thereby coupling transcription arrest to downstream repair-factor assembly. CSA, UVSSA, ubiquitin-specific protease 7 (USP7), elongation factor 1 (ELOF1), serine/threonine kinase 19 (STK19), and components of the CRL4^CSA^ complex then form a repair-coupling scaffold that coordinates ubiquitin signaling and promotes TFIIH recruitment [31,32]. TFIIH locally opens the damaged DNA, allowing XPA and RPA to verify and stabilize the lesion-containing strand [33]. XPG and XPF–ERCC1 subsequently incise the damaged strand on the 3′ and 5′ sides of the lesion, respectively. Removal of the lesion-containing oligonucleotide is followed by repair synthesis, ligation, chromatin reassembly, and restoration of transcriptional competence [34].

Although this sequential model explains the ordered recruitment and activities of TC-NER factors, it does not fully resolve the physical nature of the initiating substrate. RNAPII acts simultaneously as the sensor of transcription-blocking damage and as a large nucleoprotein obstacle that masks the damaged template strand [35].

TC-NER therefore begins not with an exposed lesion but with a chromatin-embedded elongation complex arrested at the site of damage [36]. Productive repair requires conversion of this occluded intermediate into a substrate in which the damaged strand can be opened by TFIIH, stabilized by XPA and RPA, and positioned for accurately spaced dual incision [37].

**Figure 1:**
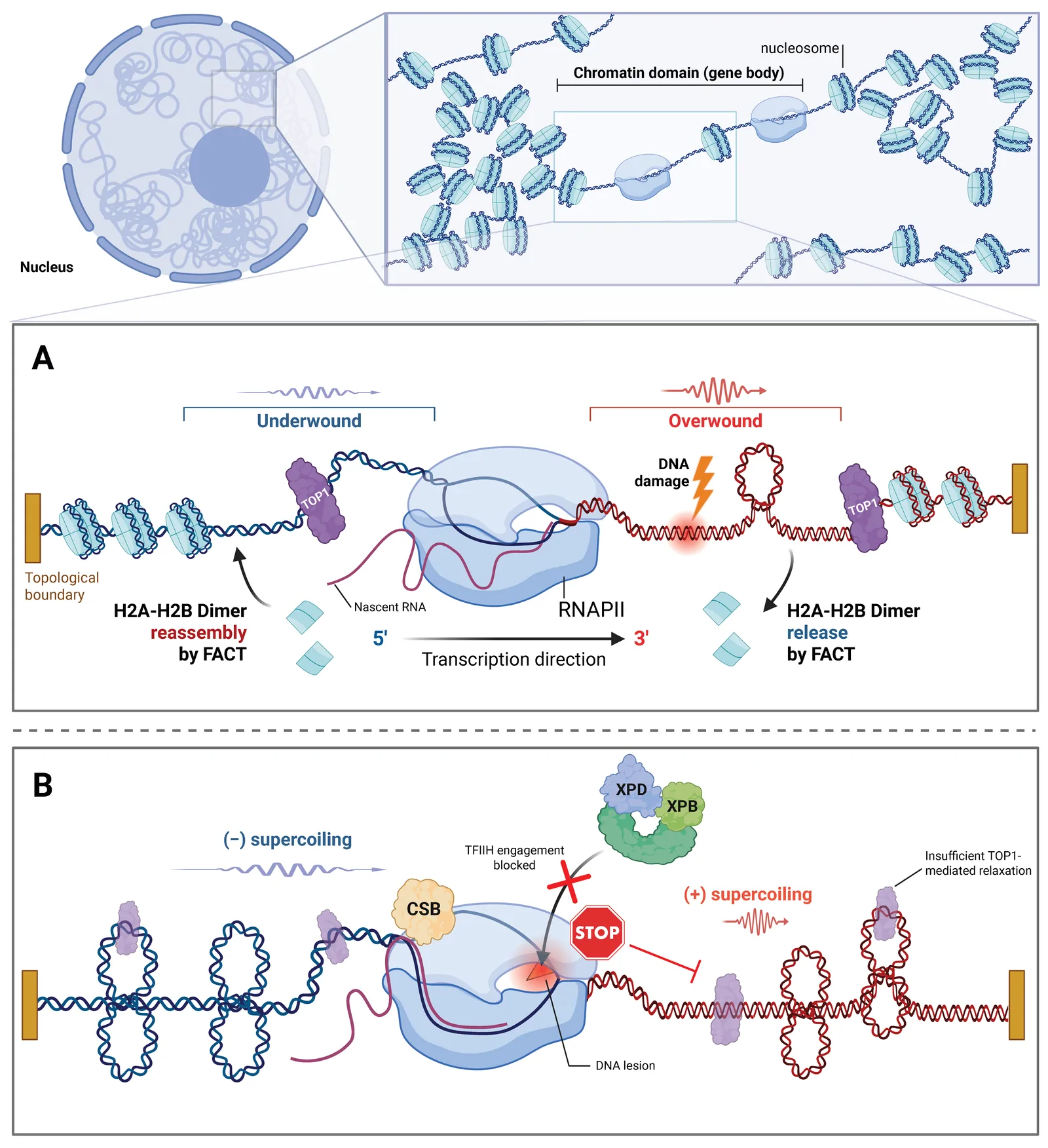
RNAPII elongation and topological constraints upon stalling. (**A**) During elongation, RNAPII traverses nucleosome-rich gene-body chromatin. Nucleosomes transiently unwrap or partially disassemble ahead of RNAPII and are reassembled behind it through histone exchange and repositioning supported by chaperones such as FACT. Elongation also generates negative supercoiling behind RNAPII and positive supercoiling ahead, both of which are relaxed by TOP1 and, depending on transcriptional output and genomic context, TOP2. At larger scales, gene bodies remain organized within local sub-TAD interaction domains rather than existing as uniformly open chromatin. (**B**) A transcription-blocking lesion arrests RNAPII and produces an RNAPII-occluded repair intermediate. Trapped positive and negative supercoiling create torsional constraints that can limit polymerase repositioning, DNA opening, and repair-factor access, whereas retained nascent RNA may favor R-loop formation. TOP1-mediated relaxation may help alleviate these constraints. Created in BioRender. Jeon, J. (2026) https://BioRender.com/b7mz955.

This conversion involves several coordinated physical and biochemical transitions (Figure 1). CSB-dependent remodeling must reorganize the arrested transcription complex and its surrounding nucleosomal environment to permit productive repair coupling [38,39]. Depending on the persistence and configuration of RNAPII arrest, the polymerase may also undergo backtracking, conformational repositioning, displacement, ubiquitylation, or proteolytic removal [40,41]. Local DNA topology represents an additional constraint because arrest can disrupt the normal dissipation of transcription-generated torsional stress, potentially allowing positive supercoiling ahead of RNAPII and negative supercoiling behind it to persist within the affected transcription unit [21,42].

Recent 5,6-dichloro-1-β-D-ribofuranosylbenzimidazole (DRB) run-off studies have added an important fate-control layer to this model by distinguishing passive transcriptional run-off from active clearance of damage-stalled RNAPII at UV-induced lesions [6]. These analyses indicate that RNAPII removal is not simply a downstream consequence of canonical NER incision. Instead, it is governed by an early hierarchical processing mechanism. CSB and CSA are required for efficient removal and degradation of stalled RNAPII, whereas loss of ELOF1, UVSSA, or STK19 primarily delays clearance [43]. By contrast, depletion of downstream NER factors involved in lesion verification or incision, including XPA, XPG, and ERCC1, does not substantially impair RNAPII removal. These observations place polymerase processing upstream of canonical dual incision and support a model in which the arrested transcription complex must be remodeled, repositioned, or, when necessary, cleared before the damaged strand becomes accessible to the core NER machinery.

## 3. A Proposed Topology-Resetting Model of TC-NER

Although actively transcribed chromatin is generally more accessible than silent chromatin [44], this accessibility does not imply a physically unconstrained or topology-free state. During elongation, RNAPII moves through nucleosome-rich gene bodies with the assistance of histone chaperones, chromatin remodelers, elongation factors, histone modifications, and topoisomerases [21,45]. These activities facilitate polymerase progression by reducing nucleosomal resistance and buffering transcription-generated torsional stress. The relevant substrate for early TC-NER is therefore transcriptionally engaged chromatin that is dynamic and partially accessible but remains mechanically constrained. Lesion-induced RNAPII arrest can disrupt this balanced state and generate a locally occluded transcription intermediate. We therefore propose that early TC-NER includes a topology-resetting process that reorganizes the arrested RNAPII complex, its nucleosomal environment, and the local torsional state to support downstream repair.

This model is based on four mechanistic principles. First, active transcription establishes a dynamic but mechanically constrained chromatin environment. Nucleosome breathing, partial DNA unwrapping, histone exchange, ATP-dependent remodeling, and topoisomerase-mediated relaxation collectively reduce barriers to elongation [46,47,48,49]. These processes buffer rather than eliminate mechanical constraints because DNA remains packaged within nucleosomes and RNAPII continuously generates torsional stress as it moves along the helical template.

Second, lesion-induced arrest can destabilize this normally buffered state. Interruption of forward translocation may restrict rotational relaxation of the RNAPII–DNA complex and allow transcription-generated torsional stress to persist locally [8,50]. Positive supercoiling ahead of the polymerase may oppose further DNA opening, whereas negative supercoiling behind it may favor local strand separation and RNA–DNA hybrid formation [51,52]. Together with neighboring nucleosomes and chromatin-associated proteins, these constraints can further reduce accessibility of the damaged template strand despite its location within an actively transcribed region.

Third, CSB-dependent remodeling and TOP1-mediated torsional regulation may functionally converge on this arrested intermediate. CSB binds lesion-stalled RNAPII and uses its ATP-dependent remodeling and scaffold functions to reorganize the RNAPII–chromatin complex into a platform compatible with TC-NER assembly [53,54]. TOP1 is not a canonical TC-NER factor, but its ability to relax transcription-generated supercoiling may help reduce torsional barriers that otherwise restrict polymerase repositioning, local DNA opening, and repair-factor access [25,55]. CSB and TOP1 may therefore influence complementary dimensions of the same substrate-conversion process, with CSB acting primarily on nucleoprotein architecture and TOP1 modulating the surrounding topological state.

Fourth, productive engagement of the canonical NER machinery is likely favored once the arrested transcription complex has acquired a sufficiently permissive structural and topological configuration. Such an intermediate would support TFIIH loading and DNA opening, XPA/RPA-mediated strand verification, XPG- and XPF–ERCC1-dependent dual incision, repair synthesis, and subsequent chromatin restoration. Repair-factor recruitment alone may therefore be insufficient when the damaged strand remains sterically occluded or mechanically constrained.

Together, these principles place early TC-NER at the intersection of RNAPII remodeling, chromatin organization, local DNA topology, and polymerase fate. The topology-resetting framework does not classify TOP1 as a canonical component of the TC-NER machinery. Instead, it proposes that transcriptional topology control may influence whether a lesion-stalled transcription complex becomes compatible with TFIIH engagement and downstream repair or progresses toward persistent arrest, R-loop formation, and RNAPII clearance.

## 4. CSB and TOP1 as Coordinated Topology Regulators

TOP1 is a type IB topoisomerase that relaxes both positive and negative DNA supercoils through a transient single-strand cleavage–rotation–religation cycle. During this reaction, TOP1 forms a covalent TOP1–DNA cleavage intermediate, permits controlled rotation of the cleaved DNA strand to dissipate torsional stress, and subsequently religates the nick to restore DNA continuity [56,57]. During elongation, RNAPII movement through the DNA helix continuously generates positive supercoiling ahead of the polymerase and negative supercoiling behind it. Positive supercoiling ahead of RNAPII may impose torsional resistance that limits forward translocation and favors polymerase pausing [58], whereas negative supercoiling behind RNAPII can facilitate local DNA strand separation and create conditions permissive for RNA–DNA hybrid formation and R-loop accumulation [59,60]. By relaxing these topological stresses, TOP1 helps maintain a chromatin environment compatible with sustained elongation.

**Figure 2:**
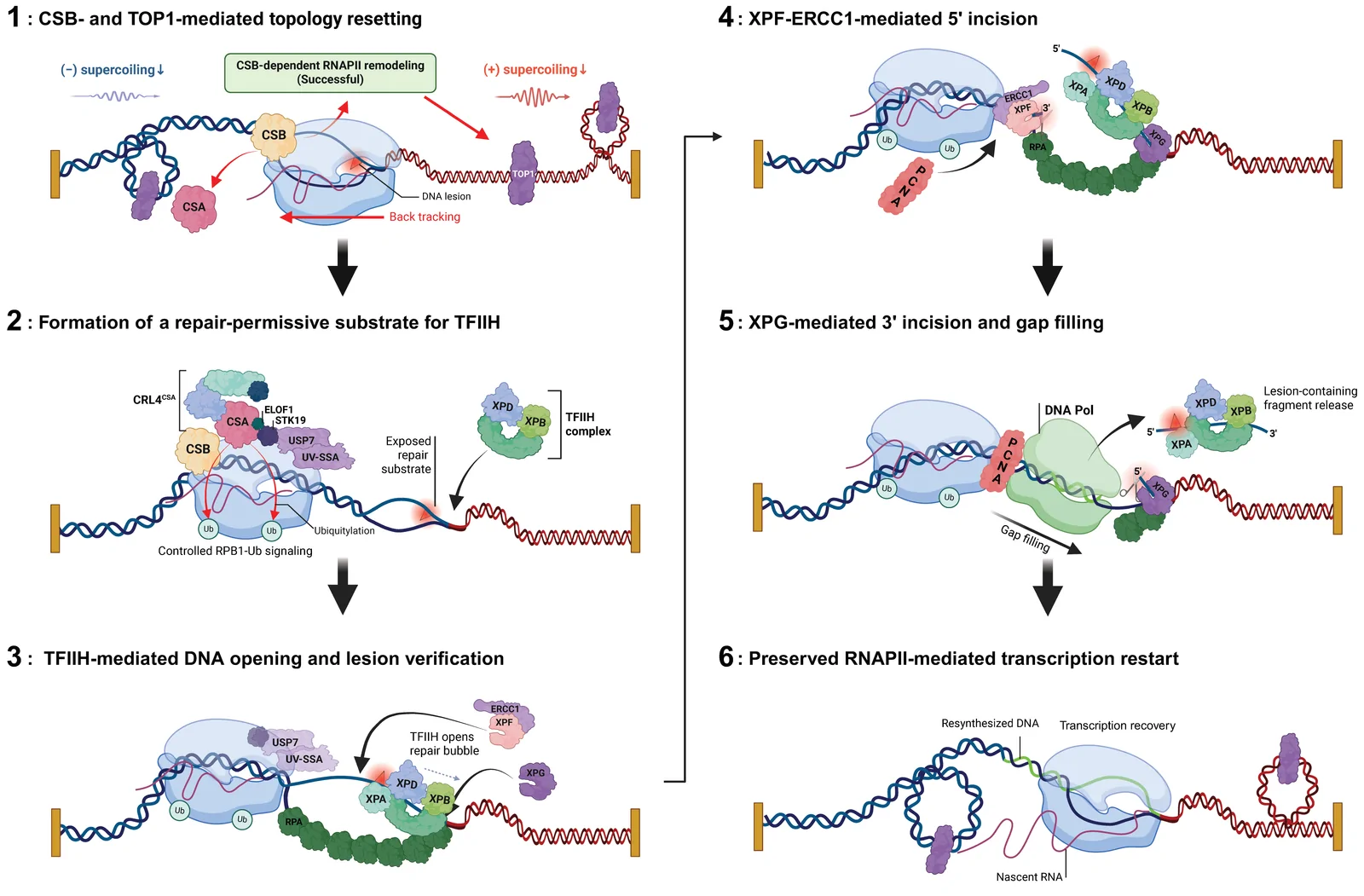
Proposed topology-permissive pathway compatible with RNAPII preservation. Topology remodeling supports TC-NER while preserving RNAPII. (**1**) CSB remodels the stalled nucleoprotein complex, whereas TOP1 relieves torsional stress by relaxing negative supercoiling behind RNAPII and positive supercoiling ahead of it. (**2**) CSA–CRL4^CSA^-dependent RPB1 ubiquitylation and the ELOF1–CSA–UVSSA–STK19 axis stabilize and position TFIIH. (**3**) TFIIH opens the damaged DNA, allowing XPA and RPA to verify the lesion-containing strand and assemble the pre-incision complex. (**4–5**) XPF–ERCC1 and XPG perform 5′ and 3′ incisions, respectively, while repair synthesis restores the excised region. (**6**) RNAPII is preserved, enabling direct transcription resumption and potentially rapid recovery. Thus, a topologically constrained, RNAPII-occluded lesion is converted into a repair-permissive substrate. Created in BioRender. Jeon, J. (2026) https://BioRender.com/udaaa33.

A central hypothesis of the topology-resetting model is that efficient early TC-NER may be facilitated by coordinated regulation of both nucleoprotein architecture and DNA topology at lesion-stalled RNAPII (Figure 2). In this framework, CSB and TOP1 are not equivalent TC-NER factors, but complementary regulators that converge on the same lesion-stalled transcription intermediate. CSB remodels the nucleoprotein architecture of the arrested RNAPII complex, whereas TOP1 may help tune the local torsional state that influences polymerase repositioning, lesion exposure, and repair-factor access. Although CSB and TOP1 have traditionally been discussed in distinct conceptual contexts—CSB as a canonical TC-NER factor and TOP1 as a transcriptional topology regulator—the topology-resetting model places their activities within a shared physical problem: converting a mechanically constrained RNAPII-arrest complex into a repair-permissive chromatin intermediate.

TOP1-dependent relaxation should therefore be considered not only at the scale of local DNA turns around stalled RNAPII, but also in relation to gene-body chromatin architecture. Transcription-linked cohesin stripes and contact islands suggest that polymerase traffic can shape sub-TAD organization, whereas nucleosome spacing and microcompartmentalization may influence local insulation, chromatin flexibility, and repair-factor accessibility [61,62]. In this context, TOP1-mediated relaxation of supercoiling at lesion-stalled RNAPII may help preserve a dynamic, repair-permissive chromatin environment and prevent the arrested transcription unit from becoming locked into a topologically constrained transcription-stress intermediate.

Although the present framework initially emphasized TOP1 because of its established role in relaxing both positive and negative transcription-generated supercoils through a strand-rotation mechanism, TOP2 enzymes may provide complementary topological regulation. TOP2A and TOP2B resolve DNA crossings through transient double-strand cleavage and strand passage, and TOP2B is particularly relevant to transcription-associated chromatin in nondividing as well as proliferating cells [63]. Genome-wide supercoiling measurements indicate that both TOP1 and TOP2B contribute to the organization of human supercoiling domains [64]. The topology-resetting model should therefore not be interpreted as requiring an exclusive role for TOP1. Rather, TOP1 and TOP2 may have partially overlapping or context-dependent functions in relieving torsional stress surrounding lesion-stalled RNAPII. Their relative contributions to TC-NER remain to be experimentally determined.

Encoded by excision repair cross-complementation group 6 (ERCC6), CSB provides a complementary activity [65]. Mutations in ERCC6 cause Cockayne syndrome, a disorder characterized by photosensitivity, developmental abnormalities, neurodegeneration, growth failure, and features of premature aging [66,67]. In TC-NER, CSB is recruited to lesion-stalled RNAPII and is essential for assembly of the downstream repair factors [68]. However, CSB should not be viewed merely as a recruitment factor. Its ATPase activity and chromatin-associated functions indicate that CSB actively remodels the stalled RNAPII–chromatin substrate, thereby helping to convert a transcription-blocked complex into a repair-permissive intermediate.

At lesion-stalled RNAPII, CSB coordinates a set of interrelated remodeling events that help convert an arrested transcription complex into a repair-competent substrate. These activities include stabilizing the stalled RNAPII complex, modulating polymerase positioning or backtracking [68,69], increasing local DNA accessibility, remodeling or repositioning nearby nucleosomes, coordinating with histone chaperone pathways [54,65], and organizing early TC-NER factor assembly [5,53]. Thus, CSB functions as an ATP-dependent remodeling hub that links RNAPII arrest to subsequent TC-NER steps. Rather than opening closed chromatin de novo, CSB acts on transcriptionally engaged chromatin that has become locally constrained by lesion-stalled RNAPII.

The functional relationship between CSB and TOP1 can be viewed as a coordinated division of labor between chromatin remodeling and topological relaxation. CSB remodels the nucleoprotein architecture of the lesion-stalled RNAPII complex, whereas TOP1 modulates the torsional state of the surrounding DNA. These activities may reinforce one another; however, this relationship should be interpreted as functional convergence rather than evidence for an established physical complex. In this model, CSB-dependent remodeling may expose or reposition DNA segments to facilitate topoisomerase access, while TOP1-dependent relaxation may lower the torsional barrier that otherwise constrains CSB-driven remodeling, RNAPII repositioning, TFIIH loading, and local DNA opening (Figure 2).

CSB also operates within a broader network of TC-NER-associated factors that determine the fate of stalled RNAPII. CSA-containing DNA damage-binding protein 1 (DDB1)– cullin 4 (CUL4) ubiquitin ligase complexes regulate RNAPII-associated ubiquitin signaling [41,70], while UVSSA and USP7 stabilize repair-coupling assemblies and facilitate TFIIH recruitment [5,31]. ELOF1 supports efficient repair of transcription-blocking lesions, and STK19 has recently emerged as a core TC-NER factor linking CSA function, UVSSA ubiquitination, TFIIH positioning, and RNAPII processing [71,72,73,74]. Poly(ADP-ribose) polymerase 1 (PARP1) may further facilitate TC-NER by stabilizing the CSB–RNAPII complex at transcription-blocking lesions and promoting local chromatin plasticity through poly(ADP-ribosyl)ation [75,76]. Together, these factors help define whether the stalled complex undergoes TC-NER, transcription restart, or RNAPII ubiquitylation and clearance, without replacing the central remodeling and topology-resetting functions of CSB and TOP1.

In the absence of direct evidence, TOP1 should not be classified as a canonical TC-NER factor. Rather, TOP1 is best viewed as a transcriptional topological regulator that may shape the physical permissiveness of the lesion-stalled transcription complex. In this model, TOP1 does not act as a classical lesion-recognition factor, but may help determine whether the damaged transcription complex can be remodeled into a repair-permissive state.

This integrated view positions CSB–TOP1 convergence as a regulatory module in early TC-NER. By linking canonical TC-NER factor recruitment to the physical state of active chromatin, this model provides a mechanistic framework for understanding how defects in CSB function or TOP1-mediated topology control could contribute to persistent transcription stress, R-loop accumulation, impaired transcription recovery, neurodegeneration, premature aging phenotypes, and altered responses to transcription-blocking chemotherapeutic agents.

It is important to distinguish this proposed role from established TC-NER mechanisms. Direct measurements of DNA supercoiling at individual lesion-stalled RNAPII complexes in mammalian cells are currently lacking, and no study has yet demonstrated that TOP1 activity is specifically required for productive TC-NER. Likewise, whether mesoscale chromatin features directly influence repair at individual transcription-blocking lesions remains to be determined. Thus, the relationships proposed here should be considered experimentally testable predictions rather than established components of the TC-NER pathway.

## 5. R-Loops as Consequences of Failed Topology Resetting

R-loops are three-stranded nucleic acid structures consisting of an RNA-DNA hybrid and a displaced single-stranded DNA strand [77]. R-loop formation is tightly coupled to DNA topology [60,78]. During RNAPII elongation, negative supercoiling generated behind the polymerase promotes local DNA strand separation, thereby increasing the likelihood that nascent RNA will hybridize with the template DNA strand [79]. Under normal transcriptional conditions, unscheduled R-loop accumulation is restrained by multiple regulatory layers, including TOP1- and TOP2-mediated supercoil relaxation, RNA processing and packaging factors, RNA/DNA helicases, ribonuclease H (RNase H) enzymes, and chromatin regulators [64,80]. Thus, R-loop homeostasis is governed not by a single pathway, but by coordinated control of transcription kinetics, RNA maturation, chromatin structure, and DNA topology.

Within the TC-NER context, lesion-induced RNAPII arrest may create a local environment that favors R-loop formation. When RNAPII stalls at a bulky transcription-blocking lesion, the nascent RNA remains associated with a paused transcription complex, while negative supercoiling behind the polymerase may become locally constrained [68,78]. If TOP1-dependent torsional relaxation is insufficient [81], or if CSB-dependent remodeling and RNAPII repositioning fail to resolve the arrested complex [68], the region behind stalled RNAPII may become increasingly prone to RNA–DNA hybrid formation.

CSB deficiency has been associated with elevated R-loop accumulation and transcription-associated genome instability [82], suggesting that CSB contributes not only to canonical TC-NER but also to broader transcriptional homeostasis [23]. The topology-resetting model offers a mechanistic explanation for this link. When CSB-dependent remodeling fails to resolve lesion-stalled RNAPII complexes, and when local torsional stress is not adequately relaxed by TOP1 or other topoisomerases, negative supercoiling and nascent RNA retention may cooperate to promote R-loop formation [81]. Persistent R-loops may, in turn, reinforce transcriptional arrest, restrict repair-factor access, and amplify DNA damage signaling [77].

Importantly, R-loop accumulation may establish a feed-forward cycle of transcription stress. Once formed behind stalled RNAPII, R-loops can reinforce a constrained chromatin and topological state, sequester RNA-processing and DNA damage response factors, and impair subsequent transcription restart. In proliferating cells, persistent R-loops can collide with replication forks, generating transcription–replication conflicts and secondary DNA lesions [83]. In post-mitotic cells, particularly neurons, unresolved R-loops may contribute to long-gene transcription defects, chronic DNA damage signaling, and cellular dysfunction [84]. This framework may help explain why CSB defects produce neurological and premature aging phenotypes that cannot be fully accounted for by defective removal of UV-induced DNA lesions alone.

R-loops should therefore be integrated into the TC-NER model as potential consequences of impaired remodeling and torsional relaxation at lesion-stalled RNAPII. When this remodeling–topology balance fails, R-loops may serve as both markers and amplifiers of defective TC-NER, thereby linking impaired chromatin-topology resetting to genome instability, failed transcription recovery, neurodegeneration, and disease-associated transcription stress.

Our previous review examined the reciprocal relationship between TC-NER and R-loop homeostasis, with emphasis on how persistent RNA–DNA hybrids contribute to genome instability [3]. The present review addresses a distinct and broader mechanistic problem: how lesion-stalled RNAPII, which functions both as a damage sensor and a steric obstacle, is converted into a substrate accessible to TFIIH and the canonical NER machinery. Accordingly, R-loops are discussed here only as one potential consequence or amplifier of unresolved transcriptional topology. The principal focus of this review is the integration of RNAPII remodeling, transcription-generated DNA supercoiling, TOP1-dependent torsional regulation, mesoscale chromatin architecture, and recently defined RNAPII-clearance mechanisms into a testable substrate-conversion framework.

Although R-loop accumulation has been linked to transcription stress and CSB deficiency, direct evidence that R-loops arising at lesion-stalled RNAPII are caused by failed topology resetting during TC-NER is currently limited. The proposed relationship between local torsional stress, R-loop formation, and repair outcome should therefore be regarded as a mechanistic hypothesis requiring direct experimental testing.

## 6. TFIIH Requires a Permissive Substrate

TFIIH occupies a central position in NER by coupling lesion recognition to local DNA opening, damage verification, and pre-incision complex assembly. In both GG-NER and TC-NER, the XPB and XPD subunits of TFIIH drive DNA unwinding and lesion verification, thereby generating the open repair bubble required for the ordered positioning of following NER factors including XPA [85]. In TC-NER, however, TFIIH faces a distinct structural challenge: unlike in GG-NER, the damaged DNA is not initially presented as a freely accessible substrate but is instead occluded within a lesion-stalled RNAPII complex [22].

The early TC-NER intermediate is occupied by lesion-stalled RNAPII together with CSB and additional regulatory factors [29]. Rather than accumulating at the lesion through a simple linear sequence, these proteins assemble into a repair-coupling scaffold that supports TFIIH positioning in an orientation compatible with damaged-strand opening and lesion verification [68,86]. The topology-resetting model adds an important physical dimension to this transition. TFIIH-mediated DNA opening is energetically and structurally constrained when the lesion-containing DNA is trapped within a torsionally stressed transcription complex [87]. Positive supercoiling ahead of stalled RNAPII can oppose local DNA unwinding and increase the energetic barrier for repair bubble formation. Negative supercoiling behind RNAPII may favor R-loop formation, generating competing RNA-DNA hybrid structures that alter local DNA accessibility and polymerase architecture [19]. In addition, nucleosomes and chromatin-associated proteins surrounding the stalled complex may restrict the conformational flexibility required for TFIIH loading and XPB/XPD-mediated DNA opening [88,89,90].

The RNAPII-clearance hierarchy assigns TFIIH an additional role beyond canonical DNA opening. Once properly recruited and positioned by UVSSA, ELOF1, and STK19, TFIIH can function through its XPD helicase activity as a key driver of rapid RNAPII dissociation from UV-damaged chromatin [6]. Within the proposed topology-resetting framework, efficient TFIIH engagement may be favored when the lesion-stalled RNAPII complex has acquired a more permissive structural and topological state. The presence of TFIIH at a stalled transcription complex is unlikely to be sufficient unless the local DNA topology and chromatin architecture permit XPB/XPD-dependent DNA opening and lesion verification. TFIIH loading therefore requires a remodeled and topologically permissive substrate generated through coordinated RNAPII processing, CSB-dependent remodeling, TOP1-mediated torsional relaxation, and TC-NER scaffold maturation.

## 7. RNAPII Fates During TC-NER

A central unresolved question in TC-NER is how cells determine the fate of lesion-stalled RNAPII. RNAPII acts both as the primary sensor of transcription-blocking lesions and as a steric barrier that occludes the damaged template strand. Its fate must therefore be coordinated with chromatin remodeling, DNA topology resetting, repair-factor assembly, and transcription recovery. From this perspective, RNAPII fate represents a biochemical–mechanical decision point: coordinated remodeling and torsional relaxation may promote repair and transcription restart, whereas unresolved torsional stress may favor R-loop accumulation, RNAPII ubiquitylation, and polymerase clearance. The stalled polymerase therefore cannot simply remain at the lesion indefinitely. It must be repositioned into a repair-permissive state, preserved for transcription restart, or removed when arrest becomes persistent and potentially deleterious.

**Figure 3:**
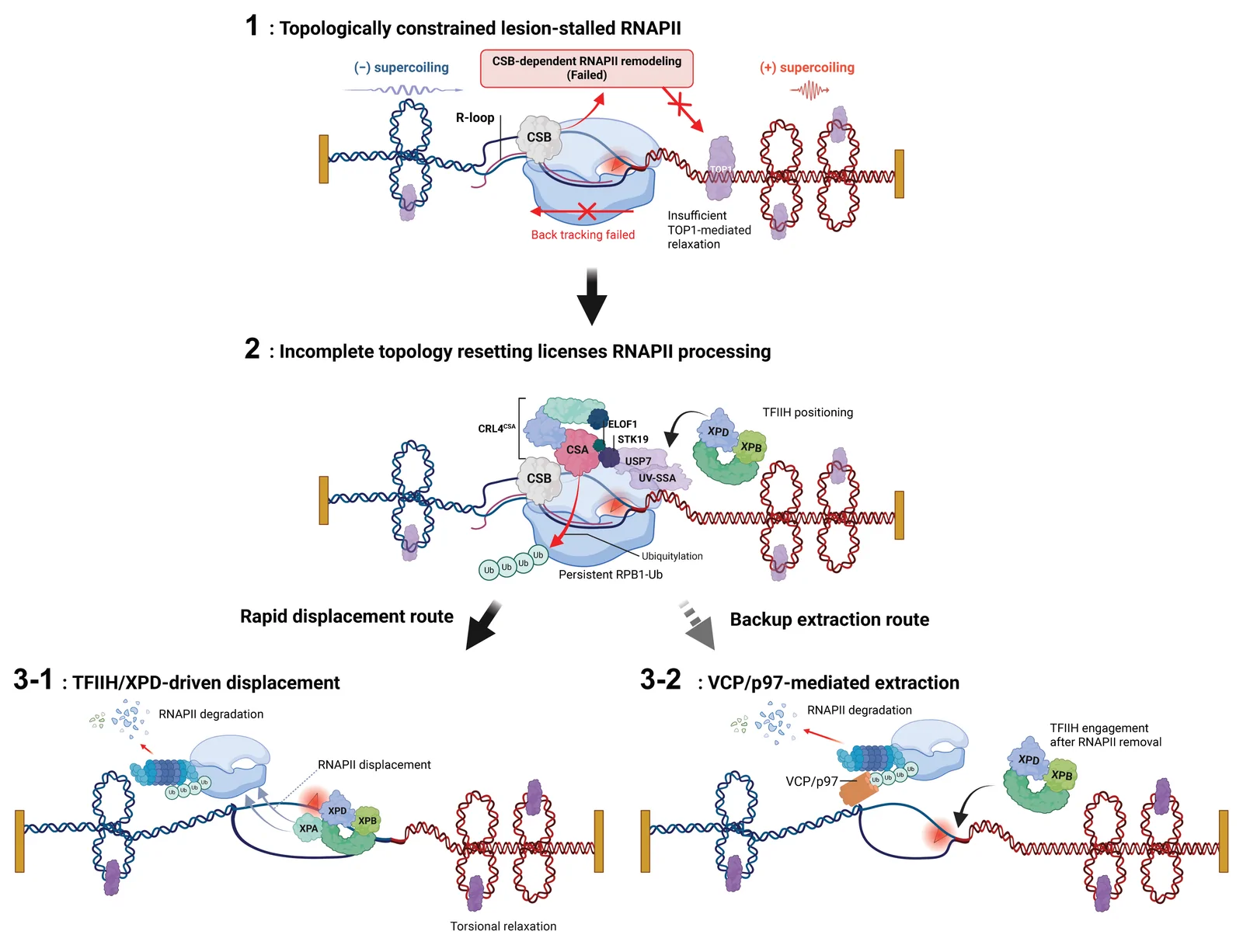
A hypothetical pathway linking unresolved topological stress to RNAPII clearance. When CSB-dependent remodeling and TOP1-mediated relaxation fail to resolve the arrested complex, lesion-stalled RNAPII is routed toward clearance rather than preservation. (**1**) Incomplete polymerase repositioning and insufficient torsional relaxation sustain local supercoiling and promote R-loop formation behind RNAPII. (**2**) CRL4^CSA^-dependent RPB1 ubiquitylation, together with the ELOF1–CSA–UVSSA–STK19 axis, promotes TFIIH recruitment and licenses RNAPII processing. (**3-1**) Persistent ubiquitylation drives TFIIH/XPD-dependent RNAPII displacement and proteasomal degradation, exposing the lesion for dual incision and repair synthesis while TOP1 relaxes residual torsional stress. (**3-2**) When TFIIH/XPD-dependent displacement is compromised, VCP/p97 acts as a slower backup extractor that removes RNAPII for proteasomal degradation. Created in BioRender. Jeon, J. (2026) https://BioRender.com/7ycucje.

Several non-mutually exclusive outcomes can be envisioned for lesion-stalled RNAPII during TC-NER (Figure 3). In one scenario, RNAPII may be preserved at or near the lesion, allowing transcription to resume after repair is completed. This outcome is likely favored when the stalled transcription complex is rapidly remodeled into a repair-permissive intermediate, torsional stress is efficiently relieved, and canonical TC-NER factors are recruited. In a second scenario, RNAPII may backtrack or undergo conformational repositioning, thereby exposing the lesion-containing DNA sufficiently to permit TFIIH loading, lesion verification, and dual incision. In a third scenario, RNAPII may remain associated with a remodeled repair complex while downstream TC-NER factors process the lesion. In a fourth scenario, persistently or irreversibly stalled RNAPII may be ubiquitylated, extracted, and ultimately degraded by the proteasome, thereby clearing the transcriptional roadblock. The selection among these possible outcomes is likely governed by the integration of multiple variables, including lesion type, the position of the lesion relative to the RNAPII active site, transcriptional activity of the affected gene, local chromatin architecture, the extent of torsional stress, R-loop formation, CSB remodeling capacity, TOP1-dependent topological relaxation, and the status of ubiquitin signaling pathways [91].

In the topology-resetting model, RNAPII fate is not determined solely by biochemical events such as factor recruitment and post-translational modification. It is also a mechanical decision shaped by the physical state of the stalled transcription complex. If CSB-dependent remodeling is successful and TOP1-dependent torsional relaxation reduces local topological constraint, the arrested RNAPII complex may be converted into a repair-permissive intermediate that supports TFIIH loading, dual incision, repair synthesis, and eventual transcription restart. In contrast, if torsional stress remains trapped, R-loops accumulate behind RNAPII, or chromatin remains locally constrained, the stalled complex may become refractory to TC-NER and instead be routed toward RNAPII ubiquitylation, VCP/p97-mediated extraction, and proteasomal clearance [36].

This decision has important biological implications. Preserving RNAPII may promote rapid transcription recovery, but only if the lesion can be made accessible without generating persistent transcription stress. Conversely, RNAPII clearance may be protective when polymerase arrest cannot be resolved, although excessive or inappropriate removal of RNAPII could compromise gene expression and delay transcription restart. Thus, TC-NER must balance two competing priorities: maintaining transcriptional continuity and preventing persistent RNAPII blockage. In the topology-resetting model, this balance is proposed to be influenced by CSB-dependent remodeling, local topological regulation, and ubiquitin-dependent RNAPII-processing pathways. Thus, RNAPII fate provides a conceptual bridge between early TC-NER assembly and late transcription recovery.

## 8. Concluding Perspective

The topology-resetting framework proposed here links RNAPII arrest to nucleosome remodeling, mesoscale chromatin architecture, R-loop control, TFIIH-dependent RNAPII clearance, canonical NER incision, transcription restart, and disease mechanisms, including Cockayne syndrome, neurodegeneration, and therapy-induced transcription stress. In this view, TC-NER depends not only on the recruitment of canonical repair factors but also on the coordinated resetting of chromatin architecture, DNA topology, and RNAPII fate across multiple length scales. Future studies combining damage-specific transcription assays, topological mapping, single-molecule chromatin profiling, and live-cell imaging will be essential to test whether topology resetting is a prerequisite for TC-NER. Such approaches may ultimately redefine TC-NER not simply as a lesion-removal pathway but as a multiscale transcription-stress resolution mechanism that coordinates DNA repair, chromatin mechanics, and RNAPII fate.

Several limitations of the topology-resetting model should be acknowledged. Direct measurements of DNA supercoiling at individual lesion-stalled mammalian RNAPII complexes are currently lacking, and TOP1 has not been established as a canonical TC-NER factor. It also remains unresolved whether the same polymerase can be retained through repair and directly resume elongation, or whether transcription recovery generally requires polymerase clearance and reinitiation. Moreover, the extent to which mesoscale chromatin features influence local TC-NER reactions has not been directly tested. The model should therefore be regarded as a testable framework that integrates established TC-NER mechanisms with predictions regarding the mechanical state of lesion-stalled chromatin.

## Data Availability

No new data were created or analyzed in this study. Data sharing is not applicable to this article.

## Abbreviations

The following abbreviations are used in this manuscript:

ADP	Adenosine diphosphate
ATP	Adenosine triphosphate
CRL4	Cullin-RING E3 ubiquitin ligase 4
CSA	Cockayne syndrome protein A
CSB	Cockayne syndrome protein B
CTCF	CCCTC-binding factor
CUL4	Cullin 4
DDB1	DNA damage-binding protein 1
DNA	Deoxyribonucleic acid
DRB	5,6-Dichloro-1-β-D-ribofuranosylbenzimidazole
E3	E3 ubiquitin ligase
ELOF1	Elongation factor 1
ERCC1	Excision repair cross-complementation group 1
ERCC6	Excision repair cross-complementation group 6
FACT	Facilitates chromatin transcription
GG-NER	Global genome nucleotide excision repair
NER	Nucleotide excision repair
PARP1	Poly(ADP-ribose) polymerase 1
RNA	Ribonucleic acid
RNAPII	RNA polymerase II
RNase H	Ribonuclease H
RPA	Replication protein A
RPB1	RNA polymerase II subunit B1
SWI2/SNF2	Switch 2/sucrose non-fermenting 2
STK19	Serine/threonine kinase 19
TAD	Topologically associating domain
TC-NER	Transcription-coupled nucleotide excision repair
TFIIH	Transcription factor IIH
TOP1	DNA topoisomerase I
TOP2	DNA topoisomerase II
USP7	Ubiquitin-specific protease 7
UV	Ultraviolet
UV-DDB	Ultraviolet-damaged DNA-binding protein complex
UVSSA	UV-stimulated scaffold protein A
VCP	Valosin-containing protein
XPA	Xeroderma pigmentosum group A protein
XPB	Xeroderma pigmentosum group B protein
XPC	Xeroderma pigmentosum group C protein
XPD	Xeroderma pigmentosum group D protein
XPF	Xeroderma pigmentosum group F protein
XPG	Xeroderma pigmentosum group G protein
